# A study of the dietary intake of Cypriot children and adolescents aged 6–18 years and the association of mother’s educational status and children’s weight status on adherence to nutritional recommendations

**DOI:** 10.1186/1471-2458-14-13

**Published:** 2014-01-08

**Authors:** Michael J Tornaritis, Elena Philippou, Charalambos Hadjigeorgiou, Yiannis A Kourides, Adamos Panayi, Savvas C Savva

**Affiliations:** 1 Research and Education Institute of Child Health, 138 Limassol Ave, 2015 Strovolos, Cyprus; 2 Pedagogical Institute of Cyprus, 40, Macedonia Ave, 2238 Latsia, Cyprus

**Keywords:** Adolescent, Child, Dietary intake, Maternal education, Weight status, Cyprus

## Abstract

**Background:**

A balanced diet is fundamental for healthy growth and development of children. The aim of this study was to document and evaluate the dietary intake of Cypriot children aged 6–18 years (y) against recommendations, and to determine whether maternal education and children’s weight status are associated with adherence to recommendations.

**Methods:**

The dietary intake of a random sample of 1414 Cypriot children was assessed using a 3-day food diary. Adherence to recommendations was estimated and the association of their mother’s education and their own weight status on adherence were explored.

**Results:**

A large percentage of children consumed less than the minimum of 45% energy (en) of carbohydrate (18.4%-66.5% in different age groups) and exceeded the recommended intakes of total fat (42.4%-83.8%), saturated fatty acids (90.4%-97.1%) and protein (65.2%-82.7%), while almost all (94.7%-100%) failed to meet the recommended fibre intake. Additionally, a large proportion of children (27.0%-59.0%) consumed >300 mg/day cholesterol and exceeded the upper limit of sodium (47.5%-78.5%). In children aged 9.0-13.9y, there was a high prevalence of inadequacy for magnesium (85.0%-89.9%), in girls aged 14.0-18.9y, of Vitamin A (25.3%), Vitamin B6 (21.0%) and iron (25.3%) and in boys of the same group, of Vitamin A (35.8%). Children whose mother was more educated were more likely to consume >15%en from protein, Odds Ratio (OR) 1.85 (95% CI:1.13-3.03) for mothers with tertiary education and exceed the consumption of 300 mg/day cholesterol (OR 2.13 (95% CI:1.29-3.50) and OR 1.84 (95% CI:1.09-3.09) for mothers with secondary and tertiary education respectively). Children whose mothers were more educated, were less likely to have Vitamin B1 (p<0.05) and Vitamin B6 intakes below the EAR (p < 0.05 for secondary school and p < 0.001 for College/University) and iron intake below the AI (p < 0.001). Overweight/obese children were more likely to consume >15%en protein (OR 1.85 (95% CI:1.26-2.71) and have a < Adequate Intake of calcium (OR 1.85 (95% CI:1.11-3.06)).

**Conclusion:**

Cypriot children consume a low quality diet. Maternal education and children’s own weight status are associated with children’s adherence to recommendations. Public health policies need to be evaluated to improve dietary quality and reduce disease burden.

## Background

A balanced diet during childhood and adolescence is crucial, both for the well-being and growth of the child [1], but also for the establishment of sound dietary habits which will persist in later life [2]. For example, adequate intakes of energy and macronutrients have essential roles in growth including muscle and brain development, while vitamins and minerals have specific individual and synergistic roles in supporting metabolic function (B Vitamins), bone mineralisation (calcium), haemoglobin production (iron) and growth (zinc). The nutrition transition, however, associated with rapid demographic and socioeconomic changes has increased the risk of obesity both in childhood [3] and in adulthood [4]. Central to this transition is the Western-type diet, characterised by excessive consumption of total and saturated fat, a high intake of sugar-sweetened beverages, as well as a low intake of fibre, legumes, fruit and vegetables which predisposes to many chronic degenerative diseases such as Type II diabetes, cardiovascular disease and some cancers in later life [5].

Cyprus, a Mediterranean country, has experienced rapid demographic and socioeconomic changes in the past 3–4 decades including a change in dietary patterns [6] which may be partly responsible for a high [7,8] and increasing prevalence of overweight (20.2%) and obesity (8.9%) in children and adolescents [9].

Undoubtedly, a child’s diet is largely influenced by their parents who provide both the genes and the eating environments (48). In particular, maternal education has been suggested as one of the key factors determining the quality of the child’s diet, with a lower educational status being associated with a worse dietary quality [10,11]. Furthermore, the role of dietary factors on obesity development during childhood is a matter still warranting further investigation [12]. To date, data on the dietary intake of children and adolescents in Cyprus have not been published.

The aim of this report was to document and evaluate the dietary intakes of Cypriot children and adolescents aged 6–18 years (y) against recommendations, and to determine whether maternal education and children’s weight status are associated with adherence to the recommendations. In this respect, the dietary intake of a random sample of Cypriot children and adolescents was assessed using a prospective dietary assessment method and compared to the USA Dietary Reference Intakes (DRIs) and the World Health Organisation/Food and Agriculture Organisation (WHO/FAO) nutritional guidelines since the European Union (EU) dietary reference values are under review [13]. The association of the maternal education and child’s weight status on adherence to recommended intakes was also estimated.

## Methods

### Study population

A school based study was performed between 2009–2010 on a nationally representative sample in elementary and secondary schools all over the non-occupied part of Cyprus, with a school class being the sampling unit. School classes were randomly selected stratifying for age, gender, district, and area of living (urban/rural) in order to include 200 participants in every 1 year age-interval for each gender. Thus 4800 children and adolescents were invited to participate taking into account, based on previous experience [8], that a large proportion would refuse participation. Since the study did not involve blood collection and was not interventional, the Cyprus Bioethical Committee following initial review advised that Bioethical approval was not necessary. Nevertheless, the study was conducted according to the guidelines laid down in the Declaration of Helsinki and written informed consent and assent were provided by the participants’ parents/guardians and the adolescents themselves before participation.

### Body measurements

Body weight (in kg) was measured after breakfast with a portable scale, with the child in light clothing and without shoes. Height (0.1 cm) was measured with a portable stadiometer at the standing position without shoes. The portable scale and stadiometer were calibrated daily. Body mass index (BMI) was calculated as weight/height^2^ (kg/m^2^). The International Obesity Task Force’s (IOTF) recommended cut-offs based on the age and sex specific values of BMI extrapolated to the adult values of 25 kg/m^2^ and 30 kg/m^2^ were used for the definition of overweight and obese children, respectively [14]. Thus appropriate age and sex-specific BMI z-scores were allocated as follows: underweight (<18.5 kg/m^2^) as −1, normal weight as: 0 (BMI: 18.5-24.9 kg/m^2^) and overweight (BMI: 25.0-30.0 kg/m^2^) and obesity (BMI ≥30 kg/m^2^) as +1 and +2, respectively.

### Collection and analysis of dietary intake data

Participants aged 12 or above or their parents/legal guardians for children aged 6-12 y were asked to record a 3-day food and beverage diary on 3 consecutive days (2 weekdays and one weekend day), to account for any differences in dietary intake between weekdays and weekends [15]. Detailed written instructions including a one-day example were provided. Participants were asked to provide recipes of foods prepared at home and were prompted to record food and beverages consumed outside the house. They were asked to report the type, brand and portion size of any pre-packaged or ready-made foods and beverages consumed. Household measures were used to report portion sizes. Food diaries were collected throughout the academic year i.e. between the months October-May. Participants with current or chronic health problems e.g. Type I diabetes, gastrointestinal disease, kidney disease, hypercholesterolemia or other disease that could have affected their dietary intake were excluded from the study. All the food diaries were analysed by an experienced dietitian using a Greek food composition software [16] in which Cypriot traditional foods and recipes analysed by the Cyprus General Laboratory [17] had been added. The average daily intake of energy, carbohydrate, protein, fat, dietary fibre and cholesterol as well as Vitamin A, Vitamin C, Vitamin B1, Vitamin B2 and Vitamin B6 and the minerals calcium, iron, phosphorus, magnesium, potassium and sodium were estimated.

Potential under-reporters and over-reporters were identified by estimating the Basal Metabolic Rate (BMR) based on the best-fitting prediction equations for Caucasians aged 5-18 y [18-20] as recently reviewed by Sabounchi *et al*[21] for the children whose weight had been measured (n = 1176; boys: 456, girls: 720). The BMR was multiplied by estimated lower and upper cut-off physical activity levels (PAL) of 1.1 and 2.2, based on the assumption of the average PAL being 1.55 following the methodology of a previous study in children [22]. The estimates of the minimum and maximum energy expenditure were then compared with reported energy intakes and participants were identified as under and over-reporters if their consumption was below the minimum or above the maximum energy expenditure respectively [23,24]. Participants identified as under-reporters or over-reporters were not excluded from analysis adopting the methodology of previous studies [25] in an attempt to reduce the possibility of introducing bias in the results [26].

### Dietary quality assessment

Dietary quality was assessed using the DRIs published by the Institute of Medicine (IoM) [27] and WHO/FAO nutritional guidelines [1,28] as described below. Participants were grouped by age using the same age-groups as the nutritional guidelines i.e. 6.0-8.9 y, 9.0-13.9 y and 14.0-18.9 y. Intakes of total fat, carbohydrate and protein expressed as percentages of total energy intake (%en) were evaluated against the acceptable macronutrient distribution ranges (AMDR) proposed by the IoM [27]. Since the AMDR for protein intake set by the IoM of 10-30%en is much higher than the 10-15%en AMDR set by the Joint WHO/FAO Expert Consultation Report on Diet, Nutrition and the Prevention of Chronic Disease [1], protein intake was also evaluated against the latter recommendation. Intakes of saturated fatty acids (SFA), monounsaturated fatty acids (MUFA) and polyunsaturated fatty acids (PUFA) expressed as %en and cholesterol intake (mg/day) were evaluated against the AMDRs and the maximum recommended daily intake respectively set by the Joint WHO/FAO Expert Consultation Report on Diet, Nutrition and the Prevention of Chronic Disease [1]. Adherence to the recommendations was assessed by determining the proportion of the population that was outside the macronutrients’ AMDR. For cholesterol, the proportion of the population that exceeded the maximum recommended intake was calculated.

The prevalence of inadequacy of micronutrient intakes was estimated using the Estimated Average Requirement (EAR) cut-point method [27]. As suggested by the IoM, the proportion of the group with intakes below the EAR can be considered similar to the proportion that does not meet the requirements [27]. For nutrients for which a tolerable upper intake level (UL) had been set, the proportion of the group with intakes above UL was also calculated [27]. For iron intake, the prevalence of inadequacy was calculated using the probability approach since the distribution of iron requirements is skewed [27]. For calcium, potassium and fibre intakes, for which EAR values have not been determined, the median intakes of the group were compared to the adequate intake (AI). As suggested by the IoM, if the mean/median intake of the group is at or above the AI, it can be assumed that the prevalence of inadequate intakes in the group is low [27]. However, if group mean/median intake is below AI, no conclusions can be drawn about the prevalence of inadequacy since the requirement distribution is not known [27]. For this reason, the proportion of the group with intakes below the AI was not calculated.

### Medical history and demographics

Using a questionnaire completed by the participants’ parents/guardians, the participants’ and their parents’ medical history including current or chronic health problems were recorded. Their parents were additionally specifically asked if they suffered from any of the following conditions: hypercholesterolaemia, hypertension, Type II diabetes mellitus, myocardial infarction, cardiovascular accident and cancer, including the type of cancer, and if yes, the age at which they were diagnosed. The participants’ (date of birth, gender, area of residence) and their family’s demographic characteristics (mother’s and father’s age, weight, height, occupation and educational level) were collected using a questionnaire.

### Residential area

The participants’ residence in urban or rural areas was assigned in accordance to the scheme used by the Statistical Service of the Ministry of Finance of Cyprus [29].

### Mother’s Education level

The mother’s self-reported education level was coded as 1: completed elementary or some secondary, 2: completed secondary school and 3: college/university education and this was considered as a proxy of the child’s socioeconomic status (SES).

### Mother’s BMI

The mother’s self reported body weight (kg) and height (m) were used to calculate BMI and classify mothers as underweight (BMI < 18.5 kg/m^2^), normal weight (BMI: 18.5-24.9 kg/m^2^), overweight (BMI: 25.0-29.9 kg/m^2^) or obese (BMI > 30 kg/m^2^) [30].

### Statistical analysis

Categorical data are presented as number and percentage and continuous data are presented as mean and standard deviation (SD). The median and interquartile range are presented for micronutrients with an AI. To determine normality of continuous variables, data were tested using the Kolmogorov-Smirnov test and by examining normality plots. The Student’s t test, for normally distributed data, the Mann–Whitney U test for non-normally distributed data and the chi-squared test for categorical data were used to compare the intakes of boys and girls of the same age group. To compare the differences in intakes over the age groups, the non-parametric Kruskal-Wallis H test was used since the two assumptions of ANOVA were not met i.e. normal distribution and homogeneity of variances. Chi-squared test was used to assess the distribution of the children’s weight status (underweight, normal weight, overweight/obese) across the three levels of maternal education.

The association of weight status and maternal education on adherence to dietary recommendations and thus dietary quality was assessed by binary logistic regression for a sub-sample of participants for which maternal education and maternal BMI had been recorded (n = 797). Normal weight children and elementary/some secondary school education were used as the reference category respectively. For assessing the association of weight status, three categories were used: (a) underweight, (b) normal weight and (c) overweight and obese children grouped together as one category. In all cases, adherence to the recommendation was coded as 0. Models were adjusted for age, gender, area of residence, mother’s BMI and under- and over-reporting, as well as mother’s education (for the weight status model only) and child’s BMI z-score (for the maternal education model only). In a further model, the interaction between mother’s education and child’s BMI were also adjusted for. Data was analysed using the Statistical Software for the Social Sciences (SPSS) Version 17.0. A p-value of ≤0.05 was considered as statistically significant.

## Results

### Response rate

A total of 3843 children and adolescents (45.5% males, 54.5% females) participated in the study of which 1414, i.e. 29.5% of the total sample, (38.6% males, 61.4% females) completed and returned a 3-day food diary. The diaries of two children were excluded due to disease: Type I diabetes (1 child) and hypercholesterolaemia (1 child). The participants’ characteristics are shown in Table 1. In comparison to the total number of children invited to participate, the study participants differed in gender (proportion of females in the total number of children invited: 54.5% vs females participating in study: 61.4%, p < 0.001) and area of residence (proportion of participants living in urban area in the total number of children: 34.8% vs study participants living in urban area: 55.4%, p < 0.001).

**Table 1:** Characteristics of the study population (n = 1414)

	**Number**	**%**
**Gender**		
Males	546	38.6
Females	868	61.4
**Area**		
Urban	783	55.4
Rural	631	44.6
**Age group**		
6.0-8.9 y	320	22.6
9.0-13.9 y	525	37.1
14.0-18.9 y	569	40.2
**Body weight classification (IOTF)**^**a**^		
Underweight	99	9.1
Normal weight	678	62.8
Overweight	210	19.4
Obese	93	8.6

^a^There were missing data on 334 participants (23.6%); percentages calculated based on the participants for whom information was available.

Anthropometric measurements were taken from a total of 1176 out of the 1414 participants (83.2%). The overall prevalence of under-reporting based on this subsample whose weight was available and thus BMR could be calculated, was 10.1% (9.9% in boys and 10.3% in girls) and over-reporting was 8.9% (3.7% in boys and 12.1% in girls, p < 0.001). Under-reporting was highest in ages 14.0-18.9y (1.6% of those aged 6.0-8.9 y, 11.5% of those aged 9.0-13.9 y and 17.8% of those aged 14.0-189 y, p < 0.001 for all comparisons). Over-reporting was more common in younger children than adolescents (13.5% of those aged 6.0-8.9 y, 5.1% of those aged 9.0-13.9 y (p < 0.001) and 3.5% of those aged 14.0-18.9 y).

An assessment of the children’s weight status by maternal education showed that there were no significant differences in the distribution of underweight, normal weight and overweight/obese children among the three levels of maternal education, chi square p value for trend p = 0.066 (Additional file 1: Table S1).

### Energy, macronutrients, fibre and cholesterol intake and comparison between genders

The daily energy, proportion of energy from macronutrients, fibre and cholesterol intakes of the participants as well as the percentages of children not meeting the recommended intakes by gender and age group are shown in Table 2.

**Table 2:** Average intakes of energy, macronutrients, fibre and cholesterol in relation to age and gender and proportion of participants not meeting the recommended intakes

	**Age group (years)**					
	**6.0-8.9**		**9.0-13.9**		**14.0-18.9**	
	**Boys**	**Girls**	**Boys**	**Girls**	**Boys**	**Girls**
	**(n = 162)**	**(n = 158)**	**(n = 211)**	**(n = 314)**	**(n = 173)**	**(n = 396)**
Energy (kcal/day)	1856 ± 273	1811 ± 272	1898 ± 337	1793 ± 341^**^	2180 ± 448	1781 ± 422^**^
Carbohydrate (%en)	48.7 ± 5.8	49.3 ± 5.2	46.5 ± 6.8	46.6 ± 6.7	42.3 ± 6.7	46.0 ± 7.2^**^
% <45/≥65% en	25.3/0.6	18.4/0	40.4/0	37.9/0.6	66.5/0	42.9^**^/0.8
Total fat (%en)	34.7 ± 5.0	34.1 ± 14.2	35.9 ± 5.5	36.6 ± 5.7	39.6 ± 5.0	37.3 ± 5.7^**^
% <25/≥35%en	2.5/43.8	1.9/42.4	1.9/59.7	2.5/61.5	0.6/83.8	2.3/66.4^**^
SFA (% en)	14.0 ± 2.7	13.7 ± 2.6	14.0 ± 2.9	14.4 ± 3.0	15.3 ± 2.9	14.3 ± 3.3^**^
% ≥10%en	92.6	90.5	92.4	93.3	97.1	90.4*
MUFA (%en)	15.2 ± 3.2	15.1 ± 2.7	16.1 ± 3.1	16.5 ± 3.5	18.1 ± 3.2	17.0 ± 3.5^**^
PUFA (%en)	5.0 ± 1.6	4.8 ± 1.4	5.4 ± 1.9	5.3 ± 1.8	5.9 ± 2.1	5.4 ± 1.8^*^
% < 6/≥10%en	81.5/1.2	86.1/0.6	68.2/3.3	72.9/1.9	58.4/2.3	68.9^*^/1.5
Protein (%en)	16.2 ± 2.5	16.2 ± 2.6	17.2 ± 3.3	16.4 ± 2.9^*^	17.7 ± 3.1	16.3 ± 3.4^**^
% < 10/≥15/≥30%en	0/68.5/0	0.6/72.8 /0	0.5/77.7/0	0.6/68.5^*^/0	0.6/82.7/0	2.3/65.2^**^/0
Fibre (g/day)	14.8 ± 5.0	14.6 ± 4.7	14.7 ± 5.0	14.0 ± 5.0	15.2 ± 6.3	14.1 ± 6.2^*^
% < 14 g/1000 kcal	100	97.8	98.6	97.8	97.7	94.7
Cholesterol (mg/day)	271.6 ± 96.7	255.3 ± 93.4	285.0 ± 108.0	269.7 ± 111.3	345.5 ± 142.6	260.0 ± 117.3^**^
% ≥300 mg/day	37.0	27.8	40.8	33.4	59.0	32.1^**^

Values presented are mean ± SD and % of participants.

%en = Percentage of total energy intake. ^*^p < 0.05, **p < 0.001 in comparison to boys of the same age-group.

In the age group 6.0-8.9 y, the intake of boys and girls was similar. There were differences, however, in the dietary intake between genders in the other age groups, with boys consuming more energy than girls in both age groups 9.0-13.9 y and 14.0-18.9 y (p < 0.001) and their diet consisting of a higher %en from protein (p < 0.05 and p < 0.001 in the age groups 9.0-13.9 y and 14.0-18.9 y respectively). Additionally, in the age group 14.0-18.9 y, the dietary intake of boys was lower in carbohydrate and higher in total fat, SFA, MUFA (p < 0.001 for all comparisons), PUFA (p < 0.05) and cholesterol (p < 0.05) as well as lower in fibre (p < 0.001) than that of girls.

### Comparison of macronutrient, cholesterol and fibre intakes with recommended intakes

As shown in Table 2, intakes of macronutrients were outside the AMDR in a large proportion of children especially those aged 14.0-18.9 y. The intake of carbohydrate was below the minimum recommended 45%en in a high percentage of children in all age-groups and particularly boys aged 14.0-18.9 y. SFA intake exceeded the maximum recommended of 10%en in the majority of children of all age groups while the intake of PUFA was lower than 6%en in a significant proportion of children. Moreover, the diet of a large proportion of children consisted of ≥15%en from protein while most children consumed less than the recommended fibre intake. Cholesterol intake exceeded the maximum of 300 mg/day in at least a third of children in all age groups while the intake of more than half of boys aged 14.0-18.9 y exceeded this limit. As shown in Table 2, boys aged 14.0-18.9 y were less likely than girls to adhere to recommendations.

### Micronutrient intake

Table 3 shows the estimated intakes of vitamins and minerals and the prevalence of children with inadequate intakes (for Vitamins A, C, B1 and B2 and iron, phosphorus and magnesium) as well as the prevalence of children whose intake exceeded the UL (for Vitamin A, magnesium and sodium) by gender and age group.

**Table 3:** Average intakes of vitamins and minerals and comparison with recommended intakes

	**Age group (years)**					
	**6.0-8.9**		**9.0-13.9**		**14.0-18.9**	
	**Boys**	**Girls**	**Boys**	**Girls**	**Boys**	**Girls**
	**(n = 162)**	**(n = 158)**	**(n = 211)**	**(n = 314)**	**(n = 173)**	**(n = 396)**
Vitamin A (μg RE/day)	958.4 ± 500.8	975.1 ± 452.3	884.6 ± 429.4	872.2 ± 502.1	978.5 ± 621.2	856.0 ± 545.4
% < EAR	1.9	1.9	15.2	12.4	35.8	25.3**
% > UL	43.8	51.9	5.2	5.7	1.2	0.8
Vitamin C (mg/day)	90.3 ± 54.3	90.8 ± 52.1	99.9 ± 65.6	86.3 ± 52.1	92.3 ± 57.7	93.2 ± 66.8
% < EAR	4.3	3.2	15.6	17.2	35.8	32.8
Vitamin B1 (mg/day)	1.4 ± 0.4	1.4 ± 0.4	1.5 ± 0.4	1.4 ± 0.5	1.8 ± 0.6	1.3 ± 0.5
% < EAR	0	0	5.2	7.0	9.8	15.4
Vitamin B2 (mg/day)	2.0 ± 0.6	1.9 ± 0.5	1.8 ± 0.5	1.8 ± 0.6	2.0 ± 0.6	1.7 ± 0.6
% < EAR	0.6	0	2.4	2.9	8.7	7.6
Vitamin B6 (mg/day)	1.5 ± 0.5	1.5 ± 0.5	2.6 ± 0.5	1.4 ± 0.5	1.7 ± 0.5	1.4 ± 0.5
% < EAR	0.6	0.6	6.2	8.6	12.7	21.0^*^
Calcium (mg/day)	956.9 ± 294.4	930.3 ± 254.0	928.9 ± 285.0	875.7 ± 275.0	1027.9 ± 358.6	858.9 ± 320.4
	895.7 (368.5)	934.6 (331.0)	914.8 (428.8)	855.7 (352.1)	1009.8 (466.8)	841.6 (427.3)
Iron (mg/day)	11.4 ± 3.3	10.9 ± 2.8	11.5 ± 2.6	10.7 ± 3.01	13.0 ± 3.6	10.4 ± 2.7
Prevalence of inadequacy (%)	2.2	2.4	2.5	5.7	6.9	25.3^**^
Phosphorus (mg/day)	1261.0 ± 242.4	1233.8 ± 230.0	1296.1 ± 282.8	1201.7 ± 269.8	1492.5 ± 366.7	1188.5 ± 310.8
% < EAR	0	0	17.5	28.3^*^	0	0
Magnesium (mg/day)	222.6 ± 56.3	218.7 ± 54.0	231.0 ± 69.3	211.6 ± 59.8	259.3 ± 77.5	213.7 ± 67.2
% < EAR	1.9	0.6	32.7	43.9*	85.0	89.9
% > UL	2.5	1.3	2.8	1.3	5.2	0.3^**^
Potassium (mg/day)	2336.7 ± 583.4	2310.7 ± 540.3	2364.2 ± 644.9	2166.3 ± 579.7	2514.7 ± 701.4	2157.5 ± 695.9
	2269.6 (762.9)	2299.0 (717.0)	2326.5 (919.4)	2139.0 (752.0)	2526.4 (1082.2)	2109.8 (936.5)
Sodium (mg/day)	2331.0 ± 566.3	2283.3 ± 538.1	2514.9 ± 617.2	2288.5 ± 593.5	2923.9 ± 856.3	2294.3 ± 685.6
% > UL	78.4	71.5	69.2	53.5^**^	76.3	47.5^**^

Values are mean ± SD or median (Interquartile Range) or % of participants.

RE = retinol equivalents, ^*^p < 0.05,**p < 0.001 in comparison to boys of the same age-group.

### Comparison of micronutrient intakes with recommended intakes

As shown in Table 3, the prevalence of inadequacy in the age group 6.0-8.9 y was low, suggesting that the needs of most children for these nutrients were met. The median potassium intake, however, was lower than the AI while sodium intake, on the other hand, exceeded the UL in about three-quarters of the children in this age group. The intake of Vitamin A in almost half the boys and more than half the girls of this age group exceeded the upper limit.

In the 9.0-13.9 y age group, although the prevalence of inadequacy of the B Vitamins and iron was low, the prevalence of children with inadequate Vitamin A, Vitamin C, phosphorus and magnesium intakes was substantial. The median intakes of calcium and potassium were below the AI but sodium intake exceeded the UL in more than half the children in this age group.

Adolescents aged 14.0-18.9 y had the highest prevalence of inadequacy compared to the other two age groups in both Vitamin A, Vitamin C, iron (in girls only) and magnesium intakes. Sodium intake exceeded the UL in a large proportion of children while the intakes of Vitamin A and magnesium were above the upper limit in only a few children of this age group. None of the children’s intakes in any age group exceeded the UL for Vitamins C and B6 or iron for which an UL has been set (results not shown).

### Comparison of adherence to recommendations for micronutrients between genders

As shown in Table 3, there were no differences between genders in relation to adherence to recommendations in the first age group. In the older children, there was a higher prevalence of inadequacy in girls than boys for phosphorus and magnesium in the age group 9.0-13.9 y (p < 0.001 for both comparisons), and for Vitamin B6 (p < 0.05) and iron (p < 0.001) in the age group 14.0-18.9 y. For Vitamin A however, the prevalence of inadequacy was higher in boys than girls in the age group 14.0-18.9 (p < 0.05). In both the 9.0-13.9 y and 14.0-18.9 y age groups, a higher percentage of boys than girls exceeded the UL for sodium (p < 0.001 for both comparisons) while in the age group 14.0-18.9 y only, a higher percentage of boys than girls exceeded the UL for magnesium (p < 0.01).

### Association of maternal education and child’s weight status on adherence to recommendations

Table 4 shows the associations between maternal education and child’s weight status on adherence to dietary recommendations. Children whose mother completed tertiary education were more likely than children whose mother was less educated to exceed the 15% recommended intake from protein (p < 0.05). Children whose mothers had completed secondary and tertiary level education were also more likely to consume more cholesterol than recommended (p < 0.05). On the other hand, children whose mothers were more educated, were less likely to have Vitamin B1 (p < 0.05) and Vitamin B6 intakes below the EAR (p < 0.05 for secondary school and p < 0.001 for College/University) and iron intake below the AI (p < 0.001). Adding an interaction term between mother’s education (as a proxy of SES) and children’s weight status showed that in most cases, the association of adherence to recommendations with mother’s education was not affected by the child’s BMI z score (data not shown).

**Table 4:** Logistic regression analysis to evaluate the association of mother’s education level and children’s weight status on children’s adherence to dietary recommendations (n = 797)

	**Mother’s education**^**a**^		**Weight status**^**b**^	
	**Secondary school**	**College/university**	**Underweight**	**Overweight/obese**
	**Adjusted model**^**c**^		**Adjusted model**^**d**^	
Carbohydrate <45%en	0.89 (0.56–1.42)	1.10 (0.67–1.78)	0.80 (0.44–1.46)	1.25 (0.88–1.77)
Total fat >35%en	0.79 (0.50–1.25)	0.65 (0.90–1.05)	0.63 (0.37–1.08)	1.21 (0.87–1.70)
SFA >10% en	0.88 (0.40–1.94)	0.94 (0.41–2.16)	0.98 (0.39–2.49)	0.97 (0.53–1.75)
PUFA <6%en	1.12 (0.68–1.85)	0.83 (0.49–1.39)	0.85 (0.45–1.61)	0.54 (0.37–1.78)
Protein >15%en	1.34 (0.87–2.20)	1.85 (1.13–3.03)*	1.10 (0.63–1.91)	1.85 (1.26–2.71)*
Fibre <14 g/1000 kcal	0.79 (0.20–3.20)	0.73 (0.16–3.34)	0.39 (0.10–1.52)	1.05 (0.31–3.52)
Cholesterol >300 mg/day	2.13 (1.29–3.50)*	1.84 (1.09–3.09)*	0.62 (0.34–1.11)	0.96 (0.68–1.34)
Vit A < EAR	1.24 (0.42–3.69)	1.82 (0.60–5.51)	0.86 (0.25–2.98)	0.49 (0.20–1.16)
Vit C < EAR	0.71 (0.40–1.25)	0.64 (0.34–1.18)	0.98 (0.43–2.25)	1.35 (0.84–2.16)
Vit B1 < EAR	0.41 (0.18–0.94)*	0.41 (0.16–1.03)	1.51 (0.48–4.77)	0.42 (0.17–1.05)
Vit B2 < EAR	0.51 (0.15–1.75)	0.61 (0.15–2.53)	3.23 (0.70–14.87)	1.26 (0.39–4.05)
Vit B6 < EAR	0.30 (0.13–0.62)**	0.41 (0.18–0.92)*	0.30 (0.06–1.48)	1.11 (0.56–2.23)
Calcium < AI	0.64 (0.30–1.37)	0.72 (0.34–1.55)	0.78 (0.36–1.65)	1.85 (1.11–3.06)*
Iron < AI	0.42 (0.26–0.67)**	0.43 (0.26–0.71)**	0.98 (0.55–1.74)	0.70 (0.48–1.02)
Magnesium < EAR	0.79 (0.43–1.45)	0.71 (0.37–1.34)	1.07 (0.51–2.24)	1.04 (0.66–1.63)
Phosphorus < EAR	0.81 (0.41–1.59)	0.84 (0.41–1.72)	0.47 (0.16–1.38)	0.76 (0.41–1.33)
Sodium > UL	1.54 (0.98–2.43)	1.31 (0.81–2.11)	0.88 (0.51–1.53)	1.14 (0.80–1.63)

Values are Odds Ratios (95% Confidence Intervals).

^a^Elementary/Some secondary education was used as the reference group. ^b^Normal weight group was used as the reference group. ^c^Adjusted for gender, age, area of residence, child’s BMI z-score, mother’s BMI and under- or over-reporting. ^d^Adjusted for gender, age, area of residence, mother’s education, mother’s BMI and under- or over-reporting. *p < 0.05, **p < 0.001.

With regards to the association of child’s weight status on adherence to recommendations, overweight and obese children were more likely to consume more than 15%en from protein (p < 0.05). Overweight and obese children were also less likely to meet the AI for calcium (p < 0.05). No significant associations were found for underweight children.

## Discussion

The present study which assessed the dietary intake of Cypriot children and adolescents for the first time showed that it is high in total fat, SFA and MUFA, protein and cholesterol, and low in carbohydrate, PUFA and fibre. Moreover, a significant proportion of children especially aged 9.0-18.9 y had low intakes of magnesium and Vitamin C and high intakes of sodium. The intake of Vitamin A exceeded the upper limit in about half the children aged 6.0-8.9 y while the intake of this vitamin was inadequate in a significant proportion of adolescents aged 14.0-18.9 y. Additionally, adolescent girls also had inadequate Vitamin B6 and iron intakes. It should be noted that it is not possible to comment on the adequacy of calcium and potassium intakes since the requirement distributions are not known and the AI has limited use in assessing nutrient intakes of groups. Even though specific food patterns were not assessed, the poor dietary quality may be related to both a high consumption of energy-dense convenience foods [31] and a low consumption of whole-grain foods [32]. It could also be speculated that the observed differences in dietary intake between genders may be due to differences in dietary behaviour such as consumption of breakfast and/or sugar-sweetened beverages as recently found in a European study [33].

This dietary pattern is a source of concern because it may predispose children to obesity, cardiovascular disease, Type II diabetes mellitus, osteoporosis and cancer [4,34], although the high MUFA intake, may have a protective effect especially during the first decades of life [35]. The high prevalence of adolescent girls who have insufficient iron intakes, is also of concern since it could put them at risk of iron-deficiency anaemia and thus associated consequences [36].

The poor dietary quality of the Cypriot children and especially adolescents was not surprising due to the increasing prevalence of obesity seen in Cyprus [9], for which an unbalanced dietary pattern is an important contributor. However, since the present study has a cross-sectional design, it is not possible to draw conclusions on the impact of the children’s dietary intake on weight status. The assessment of the association of weight status on adherence to recommendations, however, showed that overweight and obese children were more likely to exceed the recommended 15% energy from protein and less likely to meet the AI for calcium. Previous studies have shown that high total and animal protein intakes in infancy and childhood are related to obesity development in later years [37,38]. Moreover, results from cross-sectional and prospective studies in children and adolescents indicate either an inverse or neutral relationship between consumption of dairy products and/or calcium intake and body weight and body fat [39]. However, as mentioned above, no conclusions can be drawn about the adequacy of calcium intake. Attention should also be given to the high prevalence of inadequate magnesium intakes observed, since magnesium deficiency has been associated with insulin resistance in obese children [40].

Although comparisons of the macronutrient intake of different populations is difficult due to variation in survey methods and populations, total fat and MUFA intakes of Cypriot children were higher than those in the UK and Scandinavian countries but similar to Southern Mediterranean countries such as Greece, Spain and Italy [41]. The protein intake of the Cypriot children was among the highest in Europe, similar to those of French and Spanish children while the low percentage energy from carbohydrates in the older boys was similar to that of the lowest intake in European cohorts i.e. that of Spanish 8-year old children [41].

The low iron intake in adolescent girls is a common finding in other European countries [42] including the UK [43]. In particular, data from the UK suggest that a large proportion of adolescent girls have iron intakes below the lowest reference nutrient intake [43] and the HELENA study assessing iron status in European adolescents biochemically, found a higher percentage of adolescent girls than boys to be iron depleted [44]. Iron deficiency can lead to short term health effects such as physical and mental tiredness and inability to concentrate [36], which can affect daily life and school performance, but can also result in long term effects on cognitive function [45], and thus is a public health concern.

The findings of a high sodium intake and a low potassium intake are also of note since they are characteristic of diets high in salt and low in fresh fruit and vegetables and could predispose to high blood pressure and CVD [46]. In most other European surveys, sodium intake ranged from 1800–4800 mg/day in adolescents and was higher among girls than boys [41] while the potassium intake of Cypriot adolescents was lower than that estimated in other European studies [41]. There is a wide variation in Vitamin A intake (expressed as retinol equivalents (RE)) in different European studies with intakes ranging from 390–2000 μg RE/day, the lowest being in Yugoslavia and the highest in Norway, Sweden and Denmark [41]. It is noteworthy, that the comment by Lambert *et al*[41], (with reference to β-carotene intakes) that ‘within each survey intakes were similar in all age groups indicating that the younger, smaller children had greater intakes relative to their body weight’ also applies to our findings. The findings that Vitamin A exceeded the UL in a high percentage of the younger children and that a considerable percentage of adolescents did not meet their requirements should be interpreted with caution since Vitamin A distribution in food is highly variable and assessment of usual dietary intake requires the greatest number of days compared to all other nutrients and may not be captured by a 3-day diary [47]. Nevertheless, the low intake of Vitamin A by the older children should be further explored as it may be related to their low intake of fruit and vegetables and thus low β-carotene intake.

In the present study, maternal education, as a proxy of SES, was found to be related to children’s adherence to dietary recommendations with children whose mothers were more educated to be more likely to consume more than 15%en from protein and to exceed the maximum cholesterol intake, but on the other hand, to be more likely to consume adequate Vitamin B1, B6 and iron. We also found that the distribution of children based on their BMI status was similar in all three maternal education levels, indicating that maternal education is not associated with the child’s weight status although this needs to be further studied. Similarly, in a study of children in eight European countries, no association was found between SES and overweight/obesity in Cypriot children [48]. It should be noted though, that this study included only younger children (aged 2–9 y) from urban areas.

Previous studies related a lower maternal education with lower dietary quality [10,49] while a higher maternal education was related to better adherence to the Mediterranean diet pattern [50]. The reason for the variable results of the present study is not clear, but may be related to the wide age distribution of the children studied since the mother’s influence on food consumption may be different at younger ages compared to adolescence, as well as other socio-economic factors such as household income, family circumstances e.g. single-parenting, mother and father’s occupation and time spent away from home and food security previously shown to influence children’s adherence to guidelines [51]. Thus these factors need to be examined in future studies.

The present study has a number of strengths and limitations. With regards to strengths, it is the first study of the diet of Cypriot children and adolescents using a large, randomly-selected sample from all non-occupied areas of Cyprus and assessing intake using prospective diet records. The only previously published study assessed only children aged 10.7 ± 0.98 y using food frequency questionnaires [52]. Another strength is the analysis of food diaries using a Greek food composition database software enriched with local foods. Greek and Cypriot diets have many similarities due to the ethnicity of the two populations being the same and the geographical proximity of the countries. Moreover, the analysis of all the dietary records was carried out by the same researcher who was an experienced dietitian. Additionally, adherence to recommendations was evaluated using an IoM-proposed method which has also been used by previous studies [53].

The study is limited in that to the best of our efforts, a large proportion of children did not return food diaries, thus girls and urban areas were over-represented in the sample. This limitation was partly overcome by presenting the data by gender and age-groups. A further limitation of the study is that food intake was assessed using food diaries in which food portion sizes were estimated rather than weighted. However, the feasibility of using weighted dietary records in this population group has been questioned as they may be intrusive and burdensome [54]. With regards to the validity of the data collected, it is of note that under- and over-reporters were estimated based on the arbitrary assumption of a PAL of 1.55 and the cut-offs used in The Child Heart and Health Study in England (CHASE) [24] since no assessment of physical activity was carried out in our study. Overall the prevalence of underreporting was lower than that of the CHASE study where 21% of the children were identified as under-reporters [24] using the same PAL cut-offs but different equations to estimate BMR [55]. Although 17.8% of the adolescents were estimated to be under-reporting their intake, this finding was not unexpected since studies of adolescents unanimously show that underreporting is about 20% when diet records are used to assess energy intake [54,56,57]. Additionally, adolescence is a time period during which dieting may be common [58], thus it is likely that some diet records may reflect under-eating rather than under-reporting. Additionally, we have previously estimated that over 25% of Cypriot girls and 13% of boys aged 11-18y scored highly in the EAT-26 questionnaire suggesting a tendency for a disordered eating behaviour in this age-group [59]. Over-reporting, on the other hand, was slightly higher at 8.9% compared to 6.7% in the CHASE study, where the same cut-offs were used [24]. This may be due to the fact that the present study included children aged 6.0-8.9y, where over-reporting was more prevalent, whereas the CHASE study included children from 9.0 y onwards. In order to explore if this age group were indeed over-reporting or had increased energy requirements due to higher physical activity, physical activity should be assessed.

Further limitations of the present study are the lack of assessment of the participants’ intake of dietary supplements as well as the fact that the participants’ mother’s weight and height were self-reported. Nevertheless, the mother’s weight status was only included as a confounder in the analysis.

## Conclusion

In conclusion, Cypriot children and adolescents generally consume a diet of poor dietary quality characterized by higher than recommended total and SFA, protein, cholesterol and sodium and low in carbohydrate, PUFA and fibre as well as certain vitamins and minerals. MUFA consumption is moderate to high, however. This dietary pattern may predispose them to obesity, degenerative diseases and iron-deficiency anaemia in girls. Moreover overweight and obese children were more likely than normal weight children to exceed the upper 15% energy from protein and to consume less than the AI of calcium, while the mother’s educational level was related to both positive and negative associations with the children’s dietary intake, an issue which should be considered for directing nutritional advice. Future research should focus on ways of improving dietary intake and enhancing public health policies and their application both in the community in general and in schools aiming to reduce disease burden. Schools should try to adopt those European school policies that have been shown to be more effective for promoting both healthy eating and physical activity in children in which educational and environmental components are combined [60]. Furthermore, better results were shown to be achieved by computer-tailored personalized education in the classroom rather than a generic classroom curriculum. More effective environmental interventions included improved availability of physical activity opportunities in and around the school environment and improved availability or accessibility of healthy food options while at the same time restricting the availability and accessibility of unhealthy food options [60]. Following adoption of any of these policies, their application and efficacy should be explored aiming to continuously improve them. The role of protein intake on obesity development at different time periods during childhood should also be further explored.

## Competing interests

The authors declare that they have no competing interests. The study was funded by grants of the Pedagogical Institute of Cyprus and the Research and Education Institute of Child Health.

## Authors’ contributions

MT, CH, YK and SS conceived the study and were responsible for the overall running and organisation of data collection. MT obtained permission to carry out the study and contributed in obtaining funding. He overviewed data collection and critically revised the manuscript. CH also contributed in obtaining funding. SS was responsible for determining the study sample and carried out part of the analysis. EP carried out the statistical analysis of the study and prepared the manuscript. AP analysed all the food diaries. All authors critically revised the manuscript and approved the final version.

## Authors’ information

MT is a chemist, CH, YK and SS are paediatricians, EP is a clinical dietitian (RD) and AP is a dietitian.

## Pre-publication history

The pre-publication history for this paper can be accessed here:

http://www.biomedcentral.com/1471-2458/14/13/prepub

## Supplementary Material

Additional file 1 **Table S1.** Classification of children’s weight status by mother’s education. Click here for file
