# Supplementary material for: A study of the dietary intake of Cypriot children and adolescents aged 6–18 years and the association of mother’s educational status and children’s weight status on adherence to nutritional recommendations
Source: BMC Public Health. 2014 Jan 8;14:13. doi: 10.1186/1471-2458-14-13 (PMC3909376; doi:10.1186/1471-2458-14-13)
Supplement: Additional file 1 — Table S1. Classification of children’s weight status by mother’s education. [file 1471-2458-14-13-S1.docx]

**Supplementary Table 1**. Classification of children’s weight status by mother’s education

|  | **Children’s weight status** | | |
| --- | --- | --- | --- |
|  | Underweight (%) | Normal weight (%) | Overweight/Obese (%) |
| **Mother’s education** |  |  |  |
| Elementary/  Some secondary | 11.8 | 58.0 | 30.3 |
| Secondary education | 5.5 | 64.4 | 30.0 |
| Tertiary education | 10.7 | 61.8 | 27.5 |
